# Normal-Tension Glaucoma and Potential Clinical Links to Alzheimer’s Disease

**DOI:** 10.3390/jcm13071948

**Published:** 2024-03-27

**Authors:** Kathleen Ho, Nicole E. Bodi, Tasneem P. Sharma

**Affiliations:** 1 Eugene and Marilyn Glick Eye Institute, Department of Ophthalmology, Indiana University School of Medicine, Indianapolis, IN 46202, USA; katkho@iu.edu; 2 Pharmacology and Toxicology, Indiana University School of Medicine, Indianapolis, IN 46202, USA; nbodi@iu.edu; 3 Stark Neurosciences Research Institute, Indianapolis, IN 46202, USA

**Keywords:** normal tension glaucoma, Alzheimer’s Disease, epidemiology, biomarkers, cerebral, ocular, fluid dynamics

## Abstract

Glaucoma is a group of optic neuropathies and the world’s leading cause of irreversible blindness. Normal-tension glaucoma (NTG) is a subtype of glaucoma that is characterized by a typical pattern of peripheral retinal loss, in which the patient’s intraocular pressure (IOP) is considered within the normal range (<21 mmHg). Currently, the only targetable risk factor for glaucoma is lowering IOP, and patients with NTG continue to experience visual field loss after IOP-lowering treatments. This demonstrates the need for a better understanding of the pathogenesis of NTG and underlying mechanisms leading to neurodegeneration. Recent studies have found significant connections between NTG and cerebral manifestations, suggesting NTG as a neurodegenerative disease beyond the eye. Gaining a better understanding of NTG can potentially provide new Alzheimer’s Disease diagnostics capabilities. This review identifies the epidemiology, current biomarkers, altered fluid dynamics, and cerebral and ocular manifestations to examine connections and discrepancies between the mechanisms of NTG and Alzheimer’s Disease.

## 1. Background

Glaucoma is a group of optic neuropathies and the leading cause of irreversible blindness worldwide [1,2,3,4,5]. It is characterized by retinal ganglion cell (RGC) death and a distinct pattern of progressive peripheral visual field loss [3,6,7]. Primary open-angle glaucoma (POAG) is the most prevalent form of glaucoma and comprises almost 74% of all glaucoma cases [5,8]. The major risk factor for POAG is elevated intraocular pressure (IOP) [3], although a proportion of POAG patients present with the characteristic visual loss and IOP lower than 21 mmHg [9]. These patients are classified as having normal tension glaucoma (NTG), which represents 20–30% of glaucoma cases [9,10,11]. Since IOP levels lower than 21 mmHg are considered normal, these NTG patients are consequently diagnosed at later disease stages once notable visual loss has occurred [12]. Although some NTG patients benefit from therapeutics that lower IOP by 30%; in the collaborative normal-tension glaucoma study group, one-third of treated patients still experienced continued visual field loss [13]. Further, lowering IOP is currently the only targetable therapeutic for all glaucoma types [14]. This demonstrates the need for understanding IOP-independent pathological mechanisms of NTG to effectively develop neuroprotective treatments. Investigations into the nuances of NTG presents a wealth of data that NTG may have associations with another neurodegenerative disease, Alzheimer’s Disease (AD).

Recent neuroimaging studies have found that NTG neuronal degeneration expands beyond the eye and visual pathways within the brain [15,16]. These non-ocular manifestations support the hypothesis of IOP-independent mechanisms of NTG, and differing disease pathophysiology compared to POAG. The exploration of this disease within the domains of neuro-ophthalmology and neurodegenerative research has sparked significant debate and is emerging as a highly intriguing topic of global interest. Several studies have found NTG to be significantly associated with impaired cognitive functioning [17,18] and a higher incidence of AD [19,20,21,22], while others have disputed these associations [23,24]. In addition to a higher prevalence of NTG [25,26], predisposing genetic factors [27] and evidence of an association with cognitive changes [17,28] are stronger in studies with east Asians.

**Figure 1:**
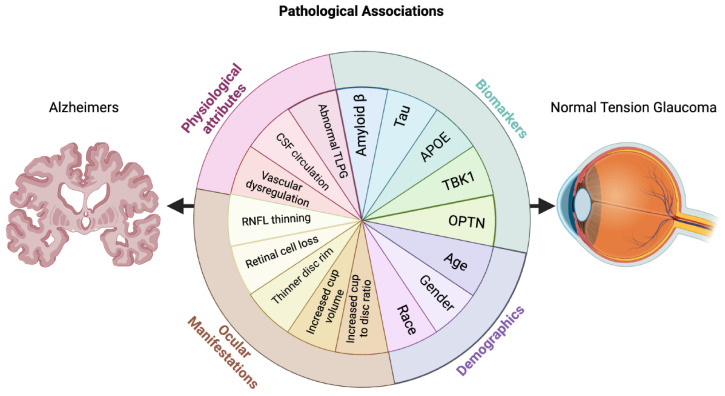
Schematic depicting the connections between Alzheimer’s Disease and normal-tension glaucoma. APOE = Apolipoprotein e4 allele, TBK1 = TANK Binding Kinase 1, OPTN = Optineurin, RNFL = retinal nerve fiber layer, CSF = cerebrospinal fluid, and TLPG = translaminar pressure gradient. Created with BioRender.com.

From the standpoint of AD, there is a growing interest in the study of ocular manifestations of AD. With a projected 150 million people living with dementia by 2050 [29], the need for early and noninvasive methods of diagnosis is integral. As an integral part of the central nervous system, the eye offers potential insights to examine cerebral neurodegeneration through retinal manifestations [30,31]. This review will describe the current knowledge on common pathophysiological mechanisms between NTG and AD, including biomarkers, cerebrospinal fluid dynamics, vascular dysfunction, cerebral and retinal changes, and the missing links between the two diseases (Figure 1).

## 2. Progressive Trends in NTG and AD Epidemiology

Both NTG and AD are progressive neurodegenerative disorders with risk factors of older age and the female sex [32]. NTG is a form of glaucoma more commonly found in the elderly, primarily because NTG often goes unnoticed until significant vision loss has occurred. Without identifiable IOP changes, it is highly underdiagnosed in younger populations and is most frequently diagnosed at approximately the age of 60 [12]. It is essential to note that this delay in diagnosis is not necessarily due to a later age of onset but rather a later age of detection [33]. On the other hand, AD typically onsets in older populations, with its prevalence increasing from 10% in those aged 65, to 40% in those over the age of 85 [34]. Visual disturbances are among the earliest reported symptoms of AD [35,36], and could serve as a potential early spectrum indicator of AD. 

Furthermore, women are disproportionately affected by both NTG [33] and AD [37]. Women are twice as likely to develop AD, and this increased risk is thought to result from a combination of factors including sex chromosomes, hormones, brain structure, and a longer average lifespan [38]. Recent research has been focusing on sex differences in microglial response to neuroinflammation and subsequent neurodegeneration [39]. Similar mechanisms may explain the higher prevalence of NTG in females as well as their increased incidence of vascular dysregulation conditions such as Raynaud’s phenomenon [40,41,42], migraines, vasospasms [43], and low blood pressure [44,45,46].

Another risk factor for NTG is race. In populations diagnosed with glaucoma, NTG is seen at the highest proportions in east Asian populations, ranging from 77% to 92% [47,48,49,50]. This is a much higher frequency compared to approximately the 30% NTG proportion seen in Caucasian POAG populations [10,51]. African populations also have a higher NTG prevalence than Caucasians, with 30–57.1% of African POAG patients diagnosed with NTG [52,53]. However, African patients still have significantly lower frequencies of NTG cases compared to Asian NTG prevalence. This suggests that race may contribute to the pathogenesis of NTG, but it remains unclear whether this association is influenced by genetic factors or environmental factors such as socioeconomic factors leading to lack of eye care. 

Beyond age, gender, and race, there are several diseases identified as potential risk factors for both NTG and AD. NTG is highly associated with systemic vascular diseases [54] including migraines [55], low systemic blood pressure [56], low diastolic ocular perfusion pressure [57], and AD [19,20,21,22,28] as mentioned previously. AD risk is also closely associated with vascular diseases [58] and vascular risk factors such as diabetes, hypertension, and dyslipidemia [59,60]. Those with migraine history have a stronger risk of developing AD than those without migraine history, with higher significance, if comorbidly obese [61]. Retinal vessel abnormalities have been found in both early stages of NTG and AD patients [21], suggesting low perfusion pressure in retinal and cerebral microvasculature [58,62] could potentially be indicative of disease pathology. 

The risk of AD in conjunction with vascular diseases [45] and vascular risk factors such as diabetes, hypertension, and dyslipidemia [46,47] is closely associated in Asian populations. A large-scale Taiwanese, retrospective cohort study included over 15,300 NTG subjects and 61,000 demographically matched subjects without glaucoma to examine the cumulative risk of AD between the two groups. The NTG group had a higher prevalence of diabetes, HTN, hyperlipidemia, coronary artery disease, and stroke, similar to AD. However, adjusting for these diseases using Cox regression still showed over 50% greater risk of developing AD in those with NTG than in comparable groups (*p* < 0.0001) [28]. A 10-year nationwide cohort study completed in Korea with 1469 NTG and 7345 controls also found a significantly higher risk of developing AD (*p* < 0.001), but not Parkinson’s disease (*p* = 0.983) [63]. Conversely, a predominantly Caucasian-based study in Denmark consisting of 69 NTG patients showed no increase in developing AD during a 12.7-year follow-up [64]. More research may be needed to examine the discrepancies seen between Asian and Caucasian NTG in conjunction with AD associations.

Data are more limited in associations between NTG and cognitive impairment. A 2021 Australasian study compared T-MoCA scores in 144 NTG and 144 high-tension glaucoma (HTG) participants over the age of 65, matched for demographics and ocular parameters. They found cognitive impairment was significantly more prevalent in the NTG cohort [17]. Another study completed in the United States found no significant difference in executive function, learning, and memory between 50 NTG and 50 control subjects aged over 50 years [23]. These conflicting findings may be due to differences in size, age group, and race/genetic makeup of the samples. To determine a true connection between cognitive impairment and NTG, future studies are needed to follow-up a large cohort with repeat testing.

## 3. Comparing Genetic Biomarkers in NTG and AD: A Comprehensive Analysis

Certain conserved biomarkers in AD and NTG could potentially indicate genetic predispositions and disease stage. These biomarkers include Amyloid b (Aβ) peptide, tau protein, Apolipoprotein (APOE), Optineurin (OPTN), and TANK Binding Kinase 1 (TBK1). Biomarkers classically associated with AD are Aβ peptide, tau protein, and APOE [65,66,67,68,69,70,71,72], while biomarkers classically associated with glaucoma include TBK1, OPTN and myocilin [73,74,75,76,77,78,79,80,81,82,83]. Detection of these biomarkers at early stages of AD or NTG is useful for both diagnostics and insight into pharmaceutical targets. Identifying correlative biomarkers could provide potential tools for early detection, treatment, and prevention of a variety of neurodegenerative diseases including AD and NTG. 

### 3.1. Insights into Aβ and Tau as Biomarkers in NTG and AD 

The relationship between Aβ and Tau with AD cases has been extensively studied; known AD pathology indicates the concurrence of extracellular deposits of Aβ peptide fibrils and intracellular neurofibrillary tangle-induced degeneration from hyperphosphorylated tau [65,66,69,70]. The amyloid hypothesis suggests that the accumulation of Aβ peptide plaque deposits cause neuronal changes and eventual cell death that is observed in AD [65,68,84]. The Aβ peptide is made from the amyloid precursor protein (APP) which is primarily found in neurons, astrocytes, microglia, endothelial cells, and meninges [85,86,87]. The APP is cleaved to produce Aβ peptide [88]. Aβ peptide residues 1-42 (Aβ42) have been found to have differing conformational states compared to Aβ peptides residues 1-40 (Aβ40), that increase their inclination towards plaque formation [68,89,90]. It is suggested that Aβ42 deposits start to occur years before the clinical signs or symptoms of AD appear [68,91]. In glaucoma, APP and the Aβ peptide can be indicative of RGC death. Under normal conditions, APP can be transported anterogradely and retrogradely between the brain and optic nerve, where it can ultimately accumulate in retinal neurons [92,93]. However, in glaucoma there is an inhibition of anterograde and retrograde transport [94,95,96]. This has been exemplified in an ocular hypertensive mouse model which showed increased levels of Aβ and APP in the pial/dural complex, the optic nerve, and the RGC layer [93]. The accumulation of APP can drive metabolic changes and alter APP homeostasis, ultimately impacting neuronal viability [93]. However, to the extent of our knowledge, there is little understanding for the APP transport in NTG models. 

### 3.2. Role of APOE4 Allele in AD and NTG: A Genetic Investigation

A factor associated with accumulation of Aβ peptides is the APOE gene e4 allele (APOE4) [97,98,99,100,101]. APOE4 does not bind as tightly to Aβ42 as the APOE e2 and 3 allelic forms, which blocks APOE4 clearance and results in its accumulation along with the Aβ peptide [68,72]. The APOE4 frequency is approximately 40% in central Africa, 24% in Malaysian aboriginals, 26% in Australia where it is especially prevalent among Australian aboriginals, and up to 28% in certain Native American tribes [100,101]. Further, the correlation between the incidence of APOE4 and AD is higher in Japanese populations compared to Caucasians [100]. In addition, a heterozygote with the APOE4 has a 3-fold greater chance of developing AD, and a homozygote has up to a 15-fold greater chance [102,103]. Alternatively, the APOE e2 allele (APOE2) has been shown to have protective effects against AD [100]. In the eye, APOE is expressed in muller cells, retinal ganglion cells, and retinal pigment epithelium of the retina [104,105]. The relationship between APOE4 and glaucoma continues to have conflicting evidence with multiple studies showing either positive or negative associations [27,106,107,108,109,110]. Evidence in support of the positive association between APOE4 and glaucoma was found in studies of Tasmanian patients with NTG and HTG. NTG patients had slightly higher frequencies of APOE4 inheritance at 38%, compared to the frequency of 34% of HTG patients with APOE4 [108]. Evidence in support of the negative association has been shown in a 2011 study of glaucoma subjects of Chinese descent. The genotyping analysis showed a decreased frequency of APOE4 in NTG patients, which lead the authors to conclude that APOE4 could have protective effects against NTG [27]. In widespread genome-wide association studies collected from the Mass Eye and Ear Infirmary and the National Eye Institute Glaucoma Human Genetics Collaboration in 2020, the findings reported that there was a statistically significant association for APOE4 with both HTG and NTG. Due to the highly significant association between APOE4 and NTG patients, the report concluded that the mechanism of APOE’s role was directly associated with retinal cells, rather than dependence on IOP changes [106]. With so many variances between publications on whether APOE4 is protective or detrimental to glaucoma development, a possible explanation of the differences in these studies can be attributed to diet. Many of the populations described to have higher frequencies of APOE4 are less predisposed to Western diets. The effects of a Western diet can result in higher cholesterol levels which both stimulate APOE activity and result in further cholesterol production in the central nervous system (CNS) [101,111]. Additionally, APOE4 is the allelic form that is least effective at exporting cholesterol, which increases the quantity of cholesterol present at the plasma membrane that can interact with the Aβ peptide, which can result in toxic Aβ accumulation [112,113]. Discrepancies between APOE regulation in AD and NTG can potentially be attributed to interaction with monocyte chemoattractant protein-1 (MCP-1/CCL2) [106]. MCP-1/CCL2 is a type of chemokine responsible for monocyte and macrophage migration and infiltration [114]. A reduction in inflammatory response within the retina of patients with the APOE4 can be protective because of decreased immune-mediated damage. However, reduced inflammation might be harmful in AD because a robust immunological reaction is required to address Aβ42-induced cytotoxicity and plaque accumulation [106,115,116]. Collectively, the identification of APOE4 indicates the likelihood of Aβ plaques that can cause AD. However, elucidating the link between APOE4 and NTG, especially in different racial groups, remains a necessity. 

Oxidative stress conditions can further modulate the activity of APOE4, which can alter its role in AD and glaucoma. APOE4 is vulnerable to the molecule 4-hydroxynonenal (4-HNE); 4-HNE is a product of lipid peroxidation under stress conditions [117,118]. Ultimately, APOE4 cannot remove 4-HNE [117,118,119]. Oxidative stress causes an upregulation of APOE4, which has inherently low antioxidant properties and is more susceptible to oxidative damage compared to the other APOE alleles [119,120,121]. This effect has been observed in human AD patients with APOE4 having higher incidences of plasma oxidation compared to other APOE alleles [104,122,123,124,125]. In AD, 4-HNE has been shown to expedite Aβ depositions and cytotoxicity. Overall assessments of AD patients’ brains show considerable levels of oxidative stress, lipid peroxidation, and low glucose metabolism [119,126,127,128,129,130,131,132]. These cellular changes are indicative of AD, which outline possible biomarkers for detection of AD [131,133]. In a study of Caucasian NTG patients there was higher total cholesterol, triglyceride, hyperlipidemia indicators, total oxidant status, and oxidative stress index levels compared to pseudoexfoliation glaucoma patients [134,135]. Hyperlipidemia can cause oxidative stress through elevated levels of reactive oxygen species (ROS), which further explains the oxidative load observed [134,136,137]. Cholesterol elevations can be explained by APOE’s role in astrocytes; APOE and cholesterol are co-secreted by astrocytes, resulting in additional neuronal secretion of cholesterol, which can cause ocular increases in cholesterol [111]. Patients with AD and NTG are both predisposed to elevated levels of oxidative stress through APOE4, which can result in metabolic- and stress-related changes that can further promote disease progression. However, especially seen in AD, this can also provide a tool to potentially predict disease in earlier stages. Early predictions of AD using oxidative stress and glucose metabolism analysis can potentially lead to identification of NTG at early stages as well. 

### 3.3. Optineurin in Focus: Bridging the Gap between NTG and AD

The combined impacts of cholesterol and oxidative stress can have inhibitory effects on autophagy that can drive AD development, specifically, with accumulation of the autophagy receptor, Optineurin (OPTN) [138]. The dysregulation of autophagy in AD and NTG indicates that OPTN can be a conserved biomarker that can be used to indicate neurodegeneration stemming from oxidative load, cholesterol levels, or neuroinflammation [77,138,139,140]. There is observed to be correlation between Aβ accumulation and increased cholesterol in the gray matter of AD brains [112,141,142,143]. In mouse models, it has been established that excess cholesterol will result in mitochondrial oxidative stress caused by excess Aβ accumulation. Experimentally, excess cholesterol loading in animal models can be accomplished by depleting the antioxidant glutathione. Depletion of glutathione has been found in AD, NTG, and POAG, indicating an increase in oxidative damage, glutamate neurotoxicity, and cholesterol [123,143,144,145,146,147,148,149,150]. The presence of the autophagy receptor, OPTN, has been found to be accumulated in aged AD models along with corresponding levels of elevated mitochondrial cholesterol levels, thus indicating that OPTN can be used to monitor the successful clearance of ROS and other cargo in AD [138]. 

In addition to the known role of OPTN in autophagy, the OPTN gene is known for its historical association with glaucoma [73,74,77]. It is believed that OPTN mutations may account for nearly 17% of hereditary forms of NTG [77]. The missense mutation of E50K to the OPTN gene is associated with autosomal dominant inheritance of early-onset familial NTG [73,151,152,153]. An additional OPTN mutation is the variant M98K, which is especially prevalent in NTG patients in Japan [153,154,155]. In vitro and in vivo models illustrate that the E50K mutation and M98K variant can result in RGC death by impaired autophagy pathways [156,157,158,159]. 

A specific relationship that works to promote autophagy is OPTN and TBK1. OPTN and TBK1 contribute to approximately 2–3% of NTG cases. Patients with these mutations usually develop severe NTG disease by age 40. The E50K mutation enhances TBK1 and OPTN binding and consequently leads to cell death [79,160]. Initiation of autophagy is caused by TBK1 phosphorylation of OPTN, promoting recruitment of MAP1LC3B (LC3B), which is required for autophagy flux [73,79,161]. A hypothesis for TBK1’s involvement in glaucoma pathogenesis is that it disrupts either autophagy or NF-kB signaling, which can lead to RGC apoptosis, further driving the glaucoma phenotype [162]. Overall, in AD and NTG, OPTN can provide evidence of dysregulated autophagy pathways that can cause accumulation of inflammatory or oxidative materials. 

## 4. Vascular Dysregulation and Impaired Pressure Dynamics in NTG and AD: A Unifying Perspective

In addition to higher sensitivity to normal IOP, three other theories aid in explaining NTG pathophysiology. These include vascular dysregulation, an abnormal translaminar pressure gradient, and impaired cerebrospinal fluid (CSF) circulation. No singular theory comprehensively explains NTG pathogenesis; therefore, it is currently believed that a combination of these pathological changes may cause glaucomatous optic disc neuropathy and RGC apoptosis. In this section, we will discuss these NTG mechanisms and their possible connections to AD pathogenesis [163].

### 4.1. Vascular Flow and the Link to NTG in AD

A leading mechanism for NTG pathogenesis is vascular dysregulation, resulting in decreasing perfusion that can contribute to retinal cell death [13,62,164,165]. Reduced blood flow to the retina, choroid, iris, and optic nerve has been examined in NTG patients [62]. Morphologically, this can be explained by significantly smaller retinal vessel diameter [166,167] and reduced density [168] in NTG patients compared to HTG patients. Similarly, early-stage AD patients are associated with retinal vessel abnormalities such as narrowed veins and decreased retinal blood flow in these veins [169]. Retinal arteriovenous nicking, consistent with narrowed veins, has been identified as predictive of long-term risk of cerebral atrophy, a risk factor for AD [170]. Suboptimal retinal vascular geometry is associated with impaired cognition [171]. Since brain microvascular abnormalities precede cognitive changes in AD, retinal vessel changes could potentially be used as indicators for cerebral microvasculature and thus early diagnosis of AD [58]. 

Another compounding issue in vascular dysregulation in AD and NTG is angiogenesis. Angiogenesis is the process in which new blood vessels are formed. Angiogenesis is physiologically vital for normal development and wound repair [172,173]. Several molecules are responsible for inducing angiogenesis, including vascular endothelial growth factor (VEGF) [173,174]. Hypoxic environments have been demonstrated to increase VEGF and activate endothelial cells [175]. This has been exemplified in the retina where mRNA expression in retinal pigment epithelial cells and retinal pericytes caused a significant increase in retinal endothelial cell growth [176]. Upregulated activation of endothelial cells contributes to a noxious environment by secretion of proteases and inflammatory factors that may promote neuronal cell death [175]. In terms of NTG, this can indicate how already demonstrated reduced blood flow to the retina and optic nerve [62], can result in ischemia that can drive VEGF activity in the retina [177]. Hypoxia-driven damage has also been observed in AD and other ocular neurodegenerative diseases, such as neovascular Age-Related Macular Degeneration (AMD). In these diseases, hypoxia can drive oxidative stress that can result in cell damage and death [178]. Additionally, VEGF has also been implicated in AD, with evidence that VEGF co-aggregates with AB plaques [179] and increased serum VEGF correlates with increased AD severity in human Apoe4 carriers [180]. In AD mouse models, VEGF paradoxically contributes to reduced cerebral blood flow, likely accelerating cognitive decline [181]. Recent studies targeting VEGF receptor-2, a more specific receptor for vascular VEGF signaling than its counterpart VEGF receptor-1 [182], show promising results in cortical capillary clearance in AD mice models [183,184]. Due to the significant impacts of VEGF, it has become a pharmacologically relevant molecule. Pharmaceutically available VEGF-inhibitors can be delivered as intravitreal injection treatments for neovascular AMD and diabetic macular edema (DME) [178,185]. A specific example of a VEGF-inhibitor that has been used in both treatment of AD and NTG is bevacizumab (Avastin) [186,187]. In an AD mouse model, Avastin was able to restore long-term memory, decrease glial activation, and reverse genetic changes associated with blood–brain barrier dysfunction, as seen in AD models [187]. Alternatively, in clinical trials using Avastin in patients with branch retinal vein occlusion with or without NTG, those with NTG had worsened visual acuity even with Avastin treatment. Further, repeated intravitreal injections of Avastin in those with AMD and DME have been shown to cause sustained increased IOP, which can increase the risk for glaucoma development or accelerate existing glaucoma [188]. Additional studies support the finding that repeated Anti-VEGF injections can lead to elevated IOP in cohorts of patients diagnosed with DME, neovascular AMD [189,190], and non-diabetic patients [191], thus indicating the complicated interplay between NTG and efficacy of anti-VEGF treatments. Furthermore, the risk for intravitreal anti-VEGF treatments extends outside of the eye and has been implicated in possible Parkinson’s-like events, hypertension-induced brain hemorrhage, and dementia [192,193,194]. Further studies examining the implication of VEGF signaling in NTG and AD are necessary to determine targeted anti-VEGF therapeutic efficacy in these diseases. 

In addition to vascular morphology changes seen in AD and NTG, decreased retinal perfusion may be due to vessel dropout. The choroid is the layer of blood vessels between the retina and sclera, providing nourishment to RGCs and regulated by the autonomic nervous system. A large-scale study found that compared to controls, the AD group had significantly thinner choroid at all analyzed points except for the temporal macula [30], which is consistent with previous studies [195,196,197,198]. Since choroid thinning is associated with aging, demographically matched control groups were a strength of the study. Similarly, NTG is associated with reduced choroid vascular index (CVI) and thickness, predominantly in Haller’s layer, the larger-sized vessel layer of the choroid [199]. There was no statistical difference between NTG and controls for CVI of choriocapillaris and Sattler’s Layer, the small-to-medium choroidal vessel layer. The breakdown of choroidal layers has not been studied for AD, to the extent of our knowledge. Of note, retinal vessel density is positively correlated with overall cognition, memory, executive function, and visual–spatial perception functions [200]. More studies are needed to compare the coincidental findings of thinning choroid vessels in both diseases and pathological implications for the eye. 

Furthermore, poor autoregulation [201,202] is more common in NTG patients than in HTG patients, causing transient episodes of ischemia and reperfusion injury [203]. This dysregulation is more evident at night when blood pressure (BP) naturally dips, further worsening low baseline diastolic BP and ocular perfusion pressure in NTG patients. Low nocturnal diastolic ocular perfusion pressure has been identified as an independent predictor of glaucomatous visual field progression in NTG patients [57]. These nocturnal pressure drops are especially evident in patients being treated for hypertension, particularly with systemic beta blockers [204]. The duration and amplitude of diastolic BP lowering during sleep are implicated in worsening of disease progression [56,205,206,207]. Poor vascular regulation is thought to influence glaucoma progression by the deprivation of RGC nutrients, increasing optic nerve sensitivity to IOP [62]. A study examined nocturnal BP variation in those with mild cognitive impairment (MCI) and normal controls, finding abnormal nocturnal BP patterns significantly higher in the MCI group [208]. MCI is defined as a transitional group between healthy aging and dementia, so early findings may be indicative of AD pathogenesis. While normal nocturnal BP dips slightly, the MCI group included significant abnormal patterns such as extreme dippers, non-dippers, and risers [208]. This suggests that abnormal autoregulation in nocturnal BP profiles is a strong indicator of MCI [208], and the extreme dipping in nocturnal pressures seen in NTG may partially explain the association of cognitive impairment with NTG [57]. Although there are differing abnormal patterns seen between MCI and NTG, the mechanism of vascular dysregulation may relate to a shared underlying pathogenesis, demonstrating the need for more research to better understand the two diseases.

Commonalities in vascular abnormalities are not limited to the eye. As mentioned, in the Section 2, both NTG and AD are associated with abnormalities in systemic vasculature and highly comorbid with systemic vascular diseases such as migraine, blood pressure dysregulation, or vascular risk factors [54,58,61]. Additionally, a prior study examined cerebral blood flow in patients with NTG and control subjects using single photon emission computed tomography scans to compare NTG perfusion patterns with AD-like vascular patterns [22]. This pattern is characteristic of lower regional cerebral blood flow from the middle cerebral artery, causing lower perfusion pressures. Although none of the patients were diagnosed with AD, 7 of the 31 NTG patients exhibited AD-like perfusion pattern, which is much higher than the 1% incidence of AD in the normal population aged 75 and over. Additionally, a 2-year follow-up period found that NTG patients with these AD-like patterns had more rapidly progressing visual field loss than those without the patterns. 

These common vascular abnormalities should be examined at the cellular level. One potential example: NTG patients have a higher prevalence of altered endothelial cell function [209,210] and increased plasma levels of endothelin-1 (ET-1) compared to controls [211,212,213]. ET-1 is a potent vasoconstrictor, mainly secreted by endothelial cells. High levels of ET-1 can induce fibrosis of vascular cells and stimulate the production of ROS [214]. However, a Japanese study showed no difference in ET-1 levels in those under the age of 60 between NTG, POAG, and healthy controls [215]. It is therefore possible that the ET-1 elevation is related to aging or later progression of disease. Interestingly, AD is also associated with an elevation in ET-1 plasma levels, and Aβ is found to significantly upregulate secretion [216]. ET-1 should be further characterized in the vascular dysregulation process by comparing NTG and AD patients at different stages of disease.

This evidence is consistent with an interaction in the pathogenesis of the two diseases; however, it is still unclear if reduced blood flow is primary or secondary to glaucomatous optic nerve damage.

### 4.2. Exploring the Relationship of the Translaminar Pressure Gradient within NTG and AD

A translaminar pressure gradient is another pathogenic element implicated in NTG. This hypothesis states that the pathogenesis of NTG is due to an abnormally high-pressure gradient across the lamina cribrosa, leading to pressure-induced neurodegeneration [217]. 

The lamina cribrosa is a sieve-like structure through which RGC axons exit the eye from the intraocular into the retrolaminar region of the optic nerve head to make direct connections to the brain. These neuronal axons are impacted by the intraocular pressure (IOP) and intracranial pressure (ICP) zones in this region which is called the translaminar pressure gradient (difference between IOP and intracranial pressure (ICP)) [218]. Thus, an increase in IOP or a decrease in ICP could potentially cause an increased pressure differential, which could result in neuronal cell damage and death. A higher translaminar gradient has been found in NTG compared to HTG and control groups [217]. This may be due to 33% lower cerebrospinal fluid pressure (CSFP) in glaucomatous individuals compared to healthy controls [219], and even lower CSFP in NTG than HTG patients [220,221]. However, meta-analysis has shown marked overlapping in lumbar CSFP measurements between NTG and healthy subjects [222]. 

A substantial proportion of AD patients have lower CSFP than controls [223]. These results potentially explain the greater risk of AD patients developing glaucoma in lieu of the translaminar pressure theory for glaucoma pathogenesis [224]. There could potentially be numerous causes of the CSFP or translaminar pressure differentials. Measuring CSFP through lumbar puncture to assess intracranial pressure is difficult as it is invasive and may not be representative of the local pressure around the lamina cribrosa. This can be evidenced by differential molecular markers, such as IgG and albumin, seen in CSF from optic nerve subarachnoid spaces compared to lumbar puncture [225]. Recent development of B-scan ultrasounds can examine optic nerve sheave diameter to successfully measure ICP noninvasively and in real time [226]. Using this technology, future studies can observe the connection of translaminar pressure gradients in NTG and AD. 

### 4.3. Altered CSF Fluid Dynamics and Bio-Active Molecules in NTG and AD Pathology

**Figure 2:**
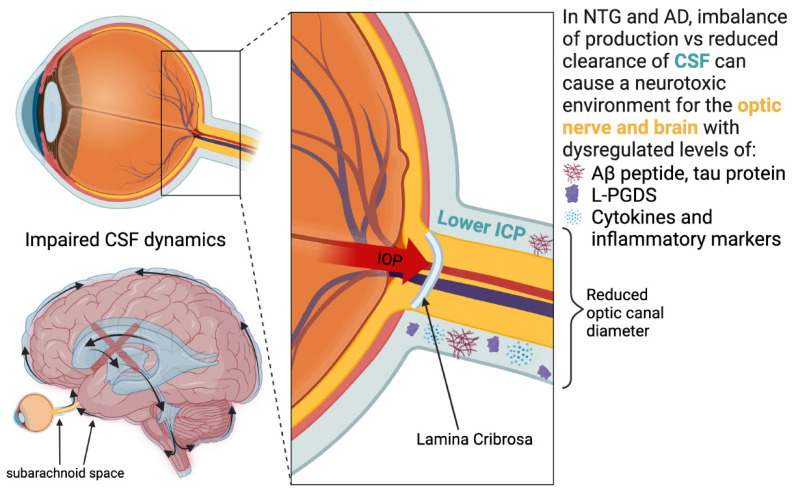
Altered CSF fluid dynamics and bio-active molecules in NTG and AD pathology. L-PGDS = lipocalin-type prostaglandin D synthases, IOP = intraocular pressure, ICP = intracranial pressure, CSF = cerebrospinal fluid, NTG = normal-tension glaucoma, AD = Alzheimer’s Disease, and Aβ = Amyloid β. Created with BioRender.com.

Another theory for NTG pathogenesis is the impairment of CSF circulation in the optic nerve sheath compartment leading to neurodegeneration [227,228]. The optic nerve is surrounded by CSF within the optic nerve sheath, making it sensitive to not only CSF pressure fluctuations, but also its contents. The CSF dynamics theory for NTG postulates reduced CSF clearance at the optic nerve leading to accumulation of neurotoxins and glaucomatous neurodegeneration [229,230]. The computed tomography cisternography imaging of the optic nerve in NTG patients showed high intracranial density of CSF in the intracranial spaces and reduced CSF turnover at the subarachnoid space of the optic nerve [231]. These findings are consistent with impaired CSF circulation, mimicking the compartment syndrome for the optic nerve. Stagnation of CSF may also play a role in the pathophysiology of neurodegenerative diseases such as AD [60,232,233]. A proposed mechanism and risk factor is reduced CSF turnover, initiating dysregulated levels of Aβ peptide and tau protein levels, and their consequential toxic molecular changes [67,234]. In NTG, a similar process is thought to occur as Aβ and tau have been identified in the CSF and deposited in the retina (Figure 2) [235,236,237,238,239]. According to this theory, glaucoma and AD neurodegeneration may occur due to an imbalance of production and clearance of these neurotoxins.

A fluid pathway closely associated with impaired CSF fluid dynamics is the glymphatic system. The glymphatic system distributes CSF throughout the brain and clears metabolites, including Aβ peptides [229,240,241,242]. Changes to the glymphatic system flow can be modulated by aquaporin (AQP) concentrations [243]. There is indication of a relationship between aquaporin-4 (AQP4) and Aβ clearance in the CSF that has been shown in rodents with depleted AQP4. The loss of AQP4 resulted in a drastic decrease in Aβ peptide clearance from the CSF and interstitial fluid [240,241,244,245,246,247]. Therefore, these results indicate that glymphatic system and concentration of AQP4 in CSF clearance can be another contributing factor to the accumulation of Aβ that is pathologically seen in AD patients. The glymphatic system has also been shown to interact with the optic nerve; AQP4s present in the optic nerve can transport CSF into the nerve. Impairment of Aβ clearance from the CSF in AD might correlate with Aβ accumulation also seen in glaucoma [222,229,247]. This indicates that overall CSF fluid dynamics connecting both the brain and eye might be integrated by AQP4 concentration in the glymphatic system.

Another cause of reduced CSF circulation may be explained by reduced optic canal diameter. In evaluations of optic canal size and severity of papilledema in intracranial idiopathic hypertension patients, the severity of papilledema modified the size of the optic canal cross-sectional area. From this study, they inferred that the optic canal diameter can influence the inflow of CSF to the subarachnoid space and impact CSF turnover [248]. This concept is applicable in NTG, which is associated with a smaller optic canal diameter than normal controls [249]. In addition to reduced CSF circulation, smaller optic nerve sheath diameter has been associated with lowered ICP [250,251]. These findings can also be integrated into the translaminar theory as history of lower ICP in NTG patients [221] due to decreased CSF density in the subarachnoid leads to an imbalance of translaminar pressure differential and eventual axonal death [231].

A closer look into the molecular changes between CSF and the optic nerve details the presence of biologically active molecules that have the potential to cause biochemical injury. A specific active molecule at high concentrations in the CSF are the beta trace proteins, which are lipocalin-type prostaglandin D synthases (L-PGDS) [225,252,253,254,255,256]. In non-glaucomatous rodent models of the eye, there is evidence of high localization of L-PGDS in the retinal pigment epithelium of the retina [257]. In the retina, it is suspected that in these cells, L-PGDS can have a role as a retinoid transporter or recycler and a lipophilic transporter [257]. In studies with NTG patients, elevated concentrations in the perioptic CSF have been found compared to patients without NTG [258]. Additionally, higher concentrations of L-PGDS have been found in the compartmentalized optic nerve CSF compared to the lumbar CSF in NTG patients with optic nerve sheath compartmentation [258]. The differential L-PGDS concentrations discovered to be secondary to CSF dynamics corroborate the findings from the computed tomography cisternography imaging of NTG patients [231]. L-PGDS’s role as a lipophilic transporter can allow it to bind and clear neurotoxic molecules, for instance Aβ and tau [259,260]. This has been exemplified in patients with idiopathic normal pressure hydrocephalus who were shown to have significant clearance relationship between total tau proteins and L-PGDS [260]. However, accumulation of L-PGDS in the blocked compartments of the CSF can cause damage to tissues in the affected area through deleterious effects on mitochondria and apoptosis-inducing properties at the optic nerve [225,261,262]. In relation to AD, the plasma of patients diagnosed with AD had greater concentrations of L-PGDS compared to controls. L-PGDS was found to have apoptotic properties due to the upregulation of inflammatory and oxidative stress factors, such as COX-2, ROS, and reduced antioxidants [263,264]. 

Another biologically active molecule in the CSF is the brain-derived neurotrophic factor (BDNF), which can connect CSF dynamics to other neurological capabilities [265]. BDNF is needed for the organization of neuronal networks and synaptic plasticity [265,266]. In non-AD human subjects, BDNF has been reported to be decreased in the CSF due to aging. Those with lower levels of BDNF in the CSF had poorer memory and diminished executive function [265]. The phenomenon of decreased CSF BDNF has been confirmed in patients with AD [266,267,268]. There are inconsistencies on whether BDNF levels in the CSF are higher or lower in females compared to males, this is interesting considering the evidence that women are more likely to develop AD [38,265,269]. However, the evidence does show a correlation between decreased body mass index and decreased plasma BDNF in females [269]. It was recently discovered that those with NTG have significantly lower systemic BDNF levels compared to HTG and controls, identifying a potential IOP-independent mechanism of pathogenesis [270] A limitation to this connection is that the BDNF levels were measured from serum and not the CSF; however, a study on rodent models suggested low BDNF plasma correlated to low brain and ocular BDNF [271]. More research is needed to elucidate whether this finding translates to human levels of BDNF, but this would explain current NTG therapeutics that are believed to improve BDNF production [272,273].The facilitative role of CSF with BDNF is still speculated [274,275]; it is plausible that loss of CSF-diffused BDNF at the optic nerve results in retinal neuron changes [275]. 

Similarly, CSF protein aggregates activate Toll-like receptors on microglial cells, promoting neuroinflammation [276]. As the first line of immune defense in the CNS, microglia will release inflammatory factors such as nitric oxide, TNFa, IL-6 and others, further activating downstream inflammatory processes [277]. Increased protein aggregates and myelin debris concentrations in the CSF and deposition in retinal tissue cause microglial cells to release cytotoxic factors which may potentiate neurodegeneration in AD and NTG [278]. Overall, neurons within the optic sheath are extremely sensitive to concentration changes in biologically active molecules in the CSF, and these effects must be further studied to fully comprehend their role in the pathogenesis of NTG. 

### 4.4. Ocular Manifestations in the Context of NTG and AD: An Investigative Analysis

**Figure 3:**
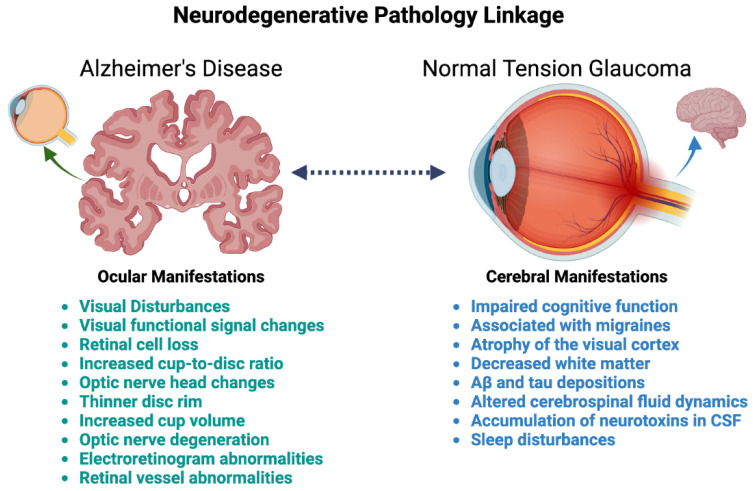
Ocular and cerebral manifestations in NTG and AD. CSF = cerebrospinal fluid and Aβ = Amyloid β. Created with BioRender.com.

Despite the varying theories primarily influencing NTG pathogenesis, several retinal manifestations are similar in NTG and AD patients (Figure 3). Optical coherence tomography (OCT) is a noninvasive imaging method that observes retinal cell degeneration through changes in macular thickness and peripapillary retinal nerve fiber layer (RNFL) [30,279,280,281,282,283]. The macular ganglion cell complex (GCC) thickness is a parameter of great interest, including the three innermost retinal layers preferentially involved in glaucoma. The GCC includes axons, cell bodies, and dendrites of RGCs from the macular RNFL, the ganglion cell layer, and the inner plexiform layer [283]. A study examined peripapillary RNFL, macular GCC, and global loss volume in NTG, AD, and healthy control subjects [284]. Global loss volume refers to the sum of negative fractional deviation in retinal thickness compared to healthy controls [285]. They found significant reductions in RNFL thickness and macular GCC, as well as significant increases in global loss volume rate in NTG and AD patients compared to controls [284]. There was no significant difference in RNFL or GCC parameters between the NTG and AD groups despite the difference in the mean age of groups as 53.6 and 73.6, respectively (*p* > 0.05) [284]. The most significant reductions in retinal thickness in AD and NTG are found in the superior-nasal quadrant, consistent with AD postmortem retinal analysis [286]. RNFL thickness is dependent on age, with 10 years of aging associated with an approximate 4µm decrease [287]. Thus, it is noteworthy that the AD groups were significantly lower compared to the control group matched for age (*p* < 0.05) [284]. This shows that the decrease in RNFL thickness in the NTG and AD groups is due to disease pathogenesis and not aging alone. Electroretinogram study has shown direct correlation between thinning of RNFL and retinal dysfunction in those with glaucoma and AD compared to controls [288]. In this study, RNFL thickness was not correlated with IOP or aging, consistent with retinal dysfunction from another mechanism causing retinal cell loss [288]. Reduced overall RNFL and GCC thickness in AD compared to controls is consistent with pathological studies [169,195,280,282,289,290,291,292,293,294,295,296,297,298,299,300]. The study also determined the duration of disease was moderately correlated with RNFL thinning, and strongly correlated with decreased GCC thickness and increased global loss volume for AD patients [284]. In this study, no significant relationship was found between mini-mental state examination (MMSE) score (a measurement of AD disease severity) and OCT parameters, suggesting that ocular changes are found early in the progression of AD [284]. This supports the use of OCT in early diagnosis of AD to assess retinal changes and determine the duration of disease. On the other hand, several studies have found a significant association between thinning in RNFL, GCC, and macula and cognitive decline [30,200,279,295,301,302,303,304,305,306,307,308,309,310,311]. Metanalysis of 25 studies comparing AD and MCI patients to healthy controls found lower peripapillary RNFL and decreased total macular thickness in AD patients [312]. These studies show the utility of OCT and OCT-Angiography in detecting early retina and capillary changes that precede cognitive changes, acting as a potential noninvasive biomarker for early AD.

The retinal cell loss and optic nerve degeneration seen in AD [286,300,313,314,315,316] has been partly implicated in visual dysfunction [317,318,319] and electroretinogram abnormalities [282,292,320] described in the clinical presentation of AD. Interestingly, retinal cell loss from Aβ deposition inside and around melanopsin RGCs (mRGCs) is thought to be implicated in sleep disturbances observed in AD as well [300]. The mRGCs intrinsically express the photopigment melanopsin and contribute to the photoentrainment of circadian rhythms through projections to suprachiasmatic nucleus [321]. Findings show a significant loss and abnormal mRGC morphology postmortem in AD compared to controls, causing circadian rhythm misalignment [300]. The variable degrees of rest and activity in circadian rhythm dysfunction in AD patients are thought to have a bidirectional relationship with pathogenesis along with many other factors [322]. The same pathophysiology in part influences NTG progression and sleep dysregulation as well [323,324,325]; however, mRGC loss is associated with the severe stages of NTG [326,327]. This is consistent with sleep disturbances in late-stage NTG and may also suggest NTG as an early spectrum of AD progression. 

There are many optic nerve head morphology patterns seen in AD that may manifest it as an ocular disease. A study of 30 AD patients found thinner disc rim, increased cup volume, and increased cup-to-disc ratio compared to age-matched controls [328]. These results correlated significantly with Alzheimer’s Disease Assessment Scale scores and longer durations of disease. Although no significant difference could be made between AD patients and controls comparing the pallor area-to-disc area ratio, AD patients with higher pallor area-to-disc ratio had higher Alzheimer’s Disease Assessment Scale scores and longer duration of disease [328]. This is suggestive that optic nerve head involvement may subgroup AD patients and be useful for monitoring progression and assessment of treatment efficacy. Additionally, a recent report demonstrated significantly thinner rim volume in NTG than HTG [329]. They also suggest many similarities between AD and NTG optic nerve patterns, including thinner disc rim, increased cup volume, and increased cup to disc ratio. More findings are needed to directly discern whether AD optic nerve manifestations are similar to NTG. 

### 4.5. A Silent Connection: Exploring Cerebral Manifestations in NTG and AD

Early-stage POAG has been associated with widespread brain abnormalities [330] including decreased anatomical connectivity through white matter tracts [331], altered resting state functional connectivity signaling [332], and grey matter atrophy [333] compared to normal controls. Although initially hypothesized to be due to pathologically high IOP and consequent retinal degeneration, recent studies have also compared HTG and NTG MRI analysis to determine if neurodegeneration beyond the eye is independent of elevated IOP [15]. A multimodal brain MRI study found functional connectivity altered in NTG at short-range visual network levels, and in HTG at long-range levels between the limbic network and secondary visual network [15]. There is a lower overall functional connectivity in NTG compared to HTG of both visual and executive networks, and increased atrophy of the visual cortex with higher axial diffusivity in HTG [15]. Lower axial diffusivity refers to decreased parallel diffusion in fiber tracts, suggestive of more axonal injury causing less coherent orientation of axons in NTG [334]. Compared to controls, NTG is also associated with decreased white matter within the corpus callosum and parietal lobe, areas beyond explanation of propagated retinal or pre-geniculate damage [16]. These findings suggest changes are, at least in part, IOP-independent and are more consistent with glaucoma as a neurodegenerative disorder. 

In AD, the brain findings are identified earliest as Aβ accumulation in the neocortex and cortical atrophy in the medial temporal region [335,336,337]. Additionally, Aβ accumulation in the hippocampus leads to hippocampal volume loss, found early in the asymptomatic and MCI stages [338]. As AD progresses, white matter [339,340,341] and grey matter [338,342] degeneration, alterations in functional connectivity [343,344,345,346,347], and visual and auditory functional MRI signal changes [348,349,350,351] have been identified. Specific connectivity pattern changes in some white and grey matter areas have been detected in AD progression [352]. These functional connectivity changes may be utilized to predict AD, but more research is needed to elucidate specific white matter connections. Functional connectivity should be compared between early AD and NTG to address any overlapping patterns. Furthermore, there are no current postmortem studies that examine the extent of AD pathology in brains of those with NTG. Given the extent of cerebral manifestations seen in NTG, this study would definitively demonstrate whether there is a correlation between the two diseases (Figure 3).

## 5. Conclusions

**Figure 4:**
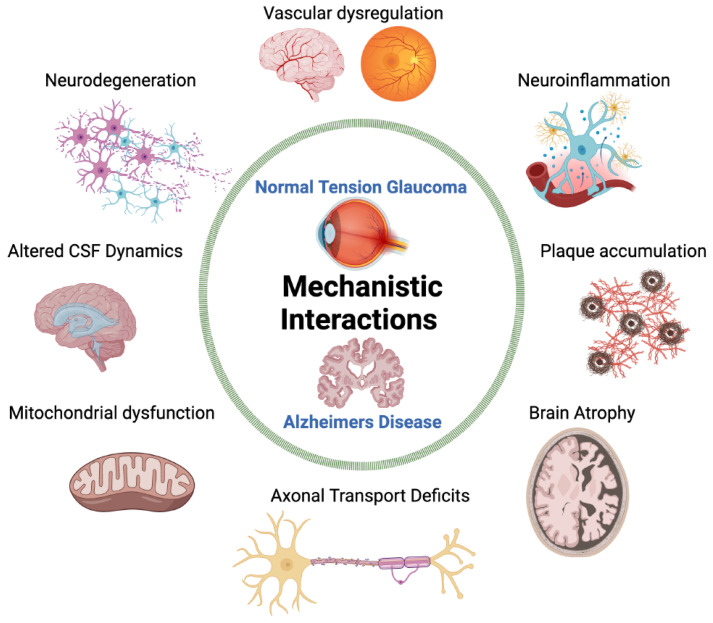
Mechanistic pathogenic linkage between Alzheimer’s and normal-tension glaucoma. CSF = cerebrospinal fluid. Created with BioRender.com.

NTG and AD are neurodegenerative diseases that may share a common mechanistic pathogenesis due to similar biomarkers, retinal manifestations, and diffuse brain atrophy (Figure 4). Vascular dysregulation, a high translaminar pressure gradient, and impaired CSF dynamics are currently research areas in proposed NTG pathogenesis. The exact mechanisms are still unclear, as none of these theories can comprehensively explain the development of the disease. New findings of Aβ and tau depositions within the vasculature, retina, and CSF may connect NTG to AD through a neuroinflammatory process. By considering the processes of neurodegeneration in AD, the pathophysiology of NTG may be better understood. 

Despite strong evidence suggesting discrete connections between NTG and AD, there are still many missing links. Future research is needed to study very low ICP groups in AD in relation to CSF dynamics and optic nerve sheath diameter. There is a need for more research on racial discrepancies seen in high proportions of the Asian population with NTG and differential retinal and cerebral manifestations. Future research should also address the age-related spectrum of disease in NTG and comorbidity with AD. Despite the efforts to slow progression of NTG with IOP-lowering treatments, no ocular medications have shown neuroprotective impact in lowering AD risk [28]. Discrepancies with nocturnal blood pressure patterns and conflicting cognitive decline association studies may suggest NTG is not an early manifestation of AD. However, the cerebral involvement of NTG cannot be ignored as it classifies NTG as a disorder beyond the eye. This review brings to light the correlative studies that identify links between NTG and AD to better understand pathogenesis and ultimately develop comprehensive therapeutics for both diseases.

## Data Availability

Data sharing is not applicable to this article as no datasets were generated or analyzed during the current study.

## Abbreviations

NTG	Normal-tension glaucoma
HTG	High-tension glaucoma
POAG	Primary open-angle glaucoma
ALS	Amyotrophic lateral sclerosis
AD	Alzheimer’s Disease
IOP	Intraocular pressure
ICP	Intracranial pressure
RGC	Retinal ganglion cell
mRGCs	Melanopsin RGCs
RNFL	Retinal nerve fiber layer
GCC	Ganglion cell complex
MMSE	Mini-mental state examination
CVI	Choroid vascular index
MCI	Mild cognitive impairment
APOE	Apolipoprotein e4 allele
OPTN	Optineurin
TBK1	TANK Binding Kinase 1
Aβ	Amyloid β
Aβ42	Aβ peptide residues 1-42
Aβ40	Aβ peptides residues 1-40
APP	Amyloid precursor protein
IL	Interleukins
TNFa	Tumor necrosis factor a
NF-kB	Nuclear factor kappa-light-chain-enhancer of activated B cells
MCP-1/CCL2	Monocyte chemoattractant protein-1
4-HNE	4-hydroxynonenal
LC3B/MAP1LC3B	Microtubule associated protein 1 light chain 3 beta
ET-1	Endothelin-1
TLPG	Translaminar pressure gradient
CSFP	Cerebrospinal fluid pressure
AQP	Aquaporin
AQP-4	Aquaporin-4
L-PGDS	Lipocalin-type prostaglandin D synthases
BDNF	Brain-derived neurotrophic factor
T-MoCA	Telephone Montreal Cognitive Assessment
